# From Metabolic Syndrome to Cardiovascular–Kidney–Metabolic Syndrome (CKM): A Clinical and Pathophysiological Continuum

**DOI:** 10.3390/biomedicines14040790

**Published:** 2026-03-30

**Authors:** Carlo Acierno, Marcello Orio, Luigi Schiavo, Angelo Saracino, Eugenio Stabile

**Affiliations:** 1 Azienda Ospedaliera Regionale San Carlo, 85100 Potenza, Italy; 2 Medical and Diabetological Center (CMSO), 84123 Salerno, Italy; marcello.orio@gmail.com; 3 Department of Medicine, Surgery and Dentistry “Scuola Medica Salernitana”, University of Salerno, 84081 Baronissi, Italy; lschiavo@unisa.it; 4 Division of Nephrology and Dialysis, San Carlo Hospital, 85100 Potenza, Italy; angelo.saracino@ospedalesancarlo.it; 5 Division of Cardiology, San Carlo Hospital, 85100 Potenza, Italy; eugenio.stabile@ospedalesancarlo.it

**Keywords:** cardiovascular–kidney–metabolic syndrome, metabolic syndrome, risk stratification, insulin resistance, chronic kidney disease, MASLD, cardiovascular disease

## Abstract

Cardiovascular–kidney–metabolic (CKM) syndrome has emerged as a broader clinical and pathophysiological framework than traditional metabolic syndrome, addressing the limitations of a purely factor-clustering approach by integrating dysfunctional adiposity, metabolic dysfunction, chronic kidney disease, and cardiovascular disease within a dynamic multiorgan continuum. This narrative review critically examines the transition from metabolic syndrome to CKM syndrome, emphasizing why the newer framework may better reflect real-world cardiorenometabolic multimorbidity and provide more clinically meaningful risk stratification through the incorporation of renal markers, subclinical cardiovascular disease, and stage-based progression. The review synthesizes the epidemiological burden of the CKM continuum and discusses the main biological mechanisms linking adipose tissue dysfunction, insulin resistance, inflammation, oxidative stress, endothelial injury, MASLD as the hepatic component of the continuum, renal vulnerability, and cardiovascular remodeling. It also considers the role of social determinants of health and the life-course perspective in shaping disease onset, progression, and access to care. Particular attention is given to the clinical implications of CKM syndrome as an interpretive and organizational model that may support earlier recognition of multiorgan risk, more integrated prevention, and less fragmented multidisciplinary management, while remaining distinct from a self-sufficient diagnostic or therapeutic algorithm. Overall, CKM syndrome should be regarded not as a new nosological entity, but as a clinically useful framework for reclassifying and managing the interconnected progression from metabolic dysfunction to renal and cardiovascular disease.

## 1. Introduction

Over the past two decades, metabolic syndrome has represented a useful clinical paradigm for describing the coexistence of visceral adiposity, arterial hypertension, atherogenic dyslipidemia, and abnormalities in glucose metabolism, providing a pragmatic tool for risk communication and for identifying individuals at greatest risk of developing type 2 diabetes mellitus and cardiovascular disease [1,2].

However, in real-world clinical practice, the course of these patients rarely fits within distinct nosological compartments [3].

More often, the metabolic, renal, and cardiovascular domains interact along a dynamic and progressive trajectory, sustained by shared and mutually reinforcing mechanisms. In this context, chronic kidney disease frequently acts as a multiplier of cardiovascular risk, whereas heart failure, especially in phenotypes associated with obesity, diabetes, and chronic nephropathy, emerges as a central manifestation of cardiorenometabolic multimorbidity [4,5].

Within this scenario, the cardiovascular–kidney–metabolic (CKM) syndrome proposed by the American Heart Association may be interpreted as a clinical-prognostic evolution of the traditional metabolic syndrome paradigm rather than as a mere terminological replacement [6,7].

Its main contribution lies in bringing dysfunctional adiposity, dysmetabolism, chronic kidney disease, and the entire spectrum of cardiovascular pathology into a unified continuum, encompassing not only atherosclerosis, but also subclinical cardiovascular disease and heart failure [6].

Compared with metabolic syndrome, this framework allows a reading of risk that is more closely aligned with clinical complexity, as it explicitly integrates renal indicators such as estimated glomerular filtration rate (eGFR) and albuminuria, highlights the role of subclinical cardiovascular damage, and situates risk within a stage-based trajectory that is potentially modifiable over time [8,9].

A further element of interest in the CKM framework is the explicit adoption of a life-course and contextual perspective. Indeed, the accumulation of metabolic, renal, and cardiovascular vulnerabilities depends not only on biological mechanisms, but is also influenced by factors such as access to care, socioeconomic context, sleep quality, the obesogenic environment, and continuity of care, all of which may affect both entry into the different stages and the speed of progression along the continuum [6,8,10].

Within the same framework, metabolic dysfunction-associated steatotic liver disease (MASLD) may be regarded as the hepatic component of the cardiorenometabolic continuum: not merely a simple comorbidity, but rather an expression of systemic metabolic dysfunction, whereas metabolic dysfunction-associated steatohepatitis (MASH) represents its inflammatory and fibro-progressive form within the same pathological spectrum [11,12].

Although it provides a clinically broader and more coherent framework than metabolic syndrome alone, the CKM model should nevertheless be interpreted with caution, taking into account its nature as an integrative framework and the need for further validation in different epidemiological settings.

In light of these considerations, the present review aims to critically re-examine the transition from metabolic syndrome to the CKM model, discussing its conceptual rationale, epidemiological burden, integrated pathophysiological mechanisms, and main clinical implications. The objective is not to introduce a new nosological label, but to assess to what extent the CKM framework allows more precise and clinically useful risk stratification, particularly through the integration of renal damage, prognostic markers such as eGFR and albuminuria, subclinical cardiovascular disease, and a longitudinal and multidimensional perspective on cardiorenometabolic multimorbidity.

## 2. The Conceptual Framework of Cardiovascular–Kidney–Metabolic Syndrome

The cardiovascular–kidney–metabolic (CKM) syndrome framework arose from the need to move beyond a fragmented interpretation of cardiometabolic multimorbidity and to bring the interaction among dysfunctional adiposity, metabolic abnormalities, chronic kidney disease, and cardiovascular disease within a unified interpretive framework [6,13].

Rather than a new nosological entity, CKM represents a clinical and prognostic model that places the patient along a dynamic continuum in which risk evolves from early dysmetabolism to overt renal and cardiovascular organ damage [6,13].

**Figure 1:**
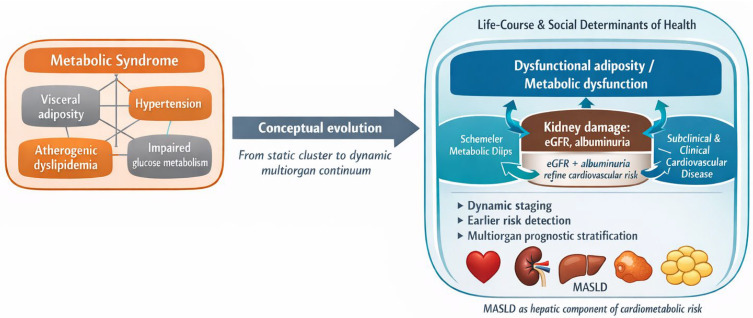
From metabolic syndrome to the cardiovascular–kidney–metabolic (CKM) continuum. The figure summarizes the shift from a clustered cardiometabolic risk-factor model to a multiorgan continuum integrating dysfunctional adiposity, kidney disease, cardiovascular involvement, and life-course determinants of risk.

As shown in Figure 1, the CKM framework moves beyond the descriptive logic of metabolic syndrome and proposes a multiorgan continuum in which dysfunctional adiposity, renal damage, and cardiovascular disease are integrated within a shared prognostic trajectory. From this perspective, eGFR, albuminuria, and MASLD contribute to an earlier and more biologically coherent definition of risk.

Within this perspective, cardiorenometabolic vulnerability is not interpreted as the sum of separate comorbidities, but rather as the expression of interdependent pathogenic circuits, also modulated over time by cumulative exposures, life transitions, and the social determinants of health [6,8,13].

Compared with metabolic syndrome, the added value of the CKM paradigm does not simply lie in expanding the list of risk factors, but in changing the interpretive logic of risk itself. Metabolic syndrome has had the merit of identifying a clinically useful cluster of visceral adiposity, arterial hypertension, atherogenic dyslipidemia, and altered glucose homeostasis; however, it remains a predominantly descriptive construct, centered on the co-occurrence of metabolic factors and less suited to representing the multiorgan trajectory of the real-world patient [2,6]. By contrast, the CKM model structurally integrates the kidney and subclinical cardiovascular disease into prognostic stratification, recognizing that risk does not merely coincide with the presence of a metabolic cluster, but rather with the progressive interaction among dysfunctional adiposity, early renal damage, cardiovascular remodeling, and subsequent clinical events [9,14,15].

In this context, the kidney is not interpreted as a late complication, but as a constitutive component of cardiorenometabolic risk. The inclusion of estimated glomerular filtration rate (eGFR) and albuminuria is one of the most distinctive features of the framework, as it allows the identification of highly vulnerable phenotypes even in the absence of overt cardiovascular disease [9,14,15].

Albuminuria, in particular, goes beyond its strictly nephrological meaning and signals a broader endothelial and microvascular dysfunction, whereas reduced eGFR identifies a phenotype with substantially higher cardiovascular and renal risk [9,14,15]. Similarly, the recognition of subclinical cardiovascular disease shifts the focus from a logic centered solely on events to one centered on progressive damage, making the model more closely aligned with pathophysiology and with the multimorbidity typically encountered in internal medicine [8,16,17].

Operationally, CKM is based on a qualitative staging system with five levels, from 0 to 4, describing the transition from the absence of cardiorenometabolic risk factors to the presence of clinically manifest cardiovascular disease [6,8,18]. Stage 1 identifies excess or dysfunctional adiposity; stage 2 includes overt metabolic risk factors and/or chronic kidney disease at moderate-to-high risk; stage 3 includes subclinical cardiovascular disease or very-high-risk phenotypes; stage 4 corresponds to clinical cardiovascular disease [6,8,18]. The meaning of staging is not merely classificatory: it translates risk into trajectory, recognizing that progression and, at least in part, regression may modify the prognostic profile over time. Within this framework, the life-course perspective and the social determinants of health are not accessory elements, but factors that influence entry into the different stages, the speed of progression, and the likelihood of early detection of damage [6,8,13,18].

**Figure 2:**
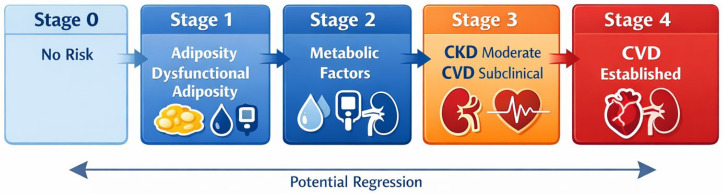
Stage-based framework of cardiovascular–kidney–metabolic (CKM) syndrome. The figure outlines the continuum from Stage 0 to Stage 4, spanning absence of risk, dysfunctional adiposity, metabolic risk factors, subclinical cardiorenal involvement, and established cardiovascular disease. It emphasizes the dynamic nature of CKM staging, including progression and the possibility of partial regression with timely risk modification and intervention.

Figure 2 conceptualizes CKM as a staged continuum, from dysfunctional adiposity and metabolic risk factors to subclinical cardiorenal involvement and overt cardiovascular disease (CVD). This framework reinforces the concept of dynamic, rather than merely descriptive, risk stratification.

This approach makes the prognostic framing of the CKM model potentially more useful than that of metabolic syndrome, because it allows an integrated interpretation of what already presents in clinical practice as a continuum: dysfunctional adiposity and dysmetabolism, metabolic dysfunction-associated steatotic liver disease, early renal involvement, subclinical cardiovascular disease, and subsequent overt clinical disease [13,17,19,20]. CKM therefore appears more consistent with the multimorbidity of the real-world patient and better suited to supporting outcome-oriented risk stratification, without being reduced to a static snapshot of coexisting factors [19,20].

Nevertheless, a cautious formulation remains appropriate: the CKM framework does not constitute a definitive taxonomy or a universally validated self-sufficient algorithm, but rather a useful clinical-conceptual framework for reorganizing multiorgan risk, to be integrated with validated predictive tools, the care context, and clinical judgment [8,20].

The main conceptual differences between metabolic syndrome and the CKM framework, together with their added clinical implications, are summarized in Table 1.

## 3. Epidemiology and Global Burden of the CKM Continuum

Cardiovascular–kidney–metabolic syndrome does not correspond to a single disease with its own autonomous incidence, but rather to a clinical framework that brings dysfunctional adiposity, dysmetabolism, chronic kidney disease, and cardiovascular disease together along a shared trajectory [6,19].

For this reason, its epidemiology should not be interpreted as the measurement of a new nosological entity, but rather as an integrated re-reading of conditions that are already highly prevalent and frequently overlapping in the general population. The main epidemiological contribution of the CKM model therefore lies in making a systemic multimorbidity readable as a multiorgan continuum, whereas it had previously often been described in separate domains [6,19,20].

The most direct data on the burden of the continuum derive from the application of CKM staging to the adult US population in National Health and Nutrition Examination Survey (NHANES) 2011–2020 [10,20].

In this analysis, only a minority of adults could be classified as stage 0, whereas the largest proportion fell within stages 1 and 2, that is, the early or intermediate phases of the continuum, already characterized by excess or dysfunctional adiposity, metabolic risk factors, and, in stage 2, also by chronic kidney disease at moderate-to-high risk [9,21].

A non-negligible proportion was also already in stages 3 and 4, corresponding respectively to subclinical cardiovascular disease or high-risk equivalents, and to clinically manifest cardiovascular disease [9,21].

Taken together, these data indicate that cardiorenometabolic vulnerability is already highly prevalent today and that a substantial part of the burden is concentrated in stages that are still potentially amenable to intensive prevention and to more accurate prognostic reclassification [8,9,10,21].

The public health relevance of the CKM continuum emerges even more clearly when the global trends of its main components are considered. Analyses from the Global Burden of Disease study document an increasing burden of overweight and obesity in adulthood, a substantial rise in the burden of diabetes mellitus between 1990 and 2021, and an equally relevant progression of chronic kidney disease at the global level [22,23,24]. From this perspective, the value of the CKM model lies not only in recording the frequency of individual highly prevalent conditions, but also in showing how these conditions converge into an integrated progression of multiorgan risk [22,23,24].

The burden, therefore, is not given by the simple sum of obesity, diabetes, chronic nephropathy, and cardiovascular disease, but rather by their biological and clinical interaction, which promotes the transition from states of early vulnerability to phenotypes of subclinical organ damage and, subsequently, to overt cardiovascular disease [19,22,23,24].

A particularly relevant aspect is that the distribution of CKM burden is not uniform either along the continuum or across different population subgroups.

The greater concentration of individuals in stages 1 and 2 indicates that a large part of risk lies in an intermediate zone in which dysfunctional adiposity, dysmetabolism, early renal abnormalities, and, in some cases, signs of initial cardiovascular involvement coexist before the onset of overt clinical events [6,10].

In this sense, the presence of chronic kidney disease, reduced estimated glomerular filtration rate, albuminuria, and subclinical cardiovascular disease contributes to defining a substantial proportion of the clinical burden because these features identify phenotypes at higher risk than would be suggested by a purely factor-centered interpretation [8,10,19,21,25].

The CKM framework therefore makes it possible to place patients along a stage-based trajectory that is useful not only descriptively, but also for prognostic stratification that is more consistent with the complexity of contemporary multimorbidity [8,10,19,21,25].

However, the epidemiological interpretation of the model requires particular caution. The prevalence of individual stages depends on the operational definitions adopted, the availability of measures such as estimated glomerular filtration rate, albuminuria, and markers of subclinical cardiovascular damage, the characteristics of the cohorts examined, and the intensity of screening for the different components of the continuum [8,21].

Moreover, most of the estimates currently available derive from US populations; consequently, these data cannot be automatically transferred to other geographical or healthcare settings without adequate external validation [8,10,19,21,25].

Differences in the prevalence of dysfunctional adiposity, diabetes, chronic kidney disease, MASLD, access to care, and the structure of healthcare systems may substantially modify both the distribution of stages and their prognostic significance.

In this regard, one of the main epidemiological limitations of the CKM framework is the need for broader validation in non-North American populations and for local calibration of the predictive tools associated with it, in order to avoid inappropriate generalizations and preserve the interpretive robustness of the model [8,25,26].

These epidemiological and prognostic considerations are synthesized in Table 2.

## 4. Integrated Pathophysiology of CKM

Cardiovascular–kidney–metabolic syndrome should be interpreted as a biologically integrated condition, rather than as the mere coexistence of obesity, diabetes, chronic kidney disease, and cardiovascular disease [13,16].

Its pathophysiological rationale lies in shared, dynamic, and self-amplifying pathogenic circuits, in which dysfunctional adiposity, insulin resistance, chronic low-grade inflammation, lipotoxicity, oxidative stress, endothelial dysfunction, neurohormonal activation, and fibroinflammatory remodeling cooperate in driving vulnerability and organ damage along a multiorgan continuum [27,28].

From this perspective, CKM describes the transition from an initial phase of metabolic-inflammatory vulnerability to a phase of subclinical damage, ultimately leading to the onset of clinically evident renal and cardiovascular manifestations [13,19].

However, this evolution does not follow a linear or rigidly ordered sequence; rather, it is configured as a network of bidirectional interactions in which adipose tissue, liver, kidney, vasculature, and myocardium act as reciprocally interconnected nodes [17,29].

It follows that risk progression does not depend on the sum of isolated organ lesions, but on feedback circuits that tend to perpetuate and amplify dysmetabolism, early renal damage, and cardiovascular remodeling, making the CKM framework more closely aligned with real-world multimorbidity than a purely cluster-based interpretation [17,29].

On this basis, the following subsections examine the main nodes of the integrated pathophysiology of CKM: dysfunctional adiposity as the biological trigger of the continuum, MASLD as the hepatic component of systemic dysfunction, the kidney as a sentinel organ and amplifier of progression, and finally cardiovascular involvement as both the subclinical and clinically manifest expression of a shared pathogenetic trajectory [16,29].

**Figure 3:**
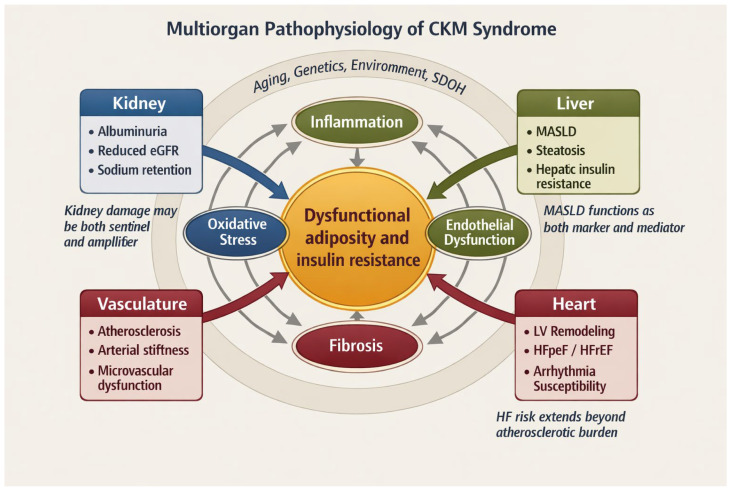
Multiorgan pathophysiology of cardiovascular–kidney–metabolic (CKM) syndrome. The figure integrates dysfunctional adiposity and insulin resistance with inflammation, oxidative stress, endothelial dysfunction, and fibrosis across the kidney, liver, vasculature, and heart. It emphasizes CKM as a self-reinforcing multiorgan network in which shared mechanisms converge on progressive organ vulnerability.

As illustrated in Figure 3, the pathophysiology of CKM is organized around a central core of dysfunctional adiposity and insulin resistance, from which shared mechanisms of inflammation, oxidative stress, endothelial dysfunction, and fibrosis radiate. This framework clarifies why CKM risk should be interpreted from a multiorgan perspective rather than as the simple coexistence of metabolic, renal, and cardiovascular abnormalities.

### 4.1. Dysfunctional Adiposity

Dysfunctional adiposity represents one of the main biological drivers of the cardiovascular–kidney–metabolic continuum and is a more informative construct than obesity defined solely on an anthropometric basis, because it reflects not only excess adipose mass, but above all the qualitative alteration of adipose tissue, its distribution, and its systemic biological activity [13,30]. From this perspective, the central pathogenetic node does not simply coincide with the amount of body fat, but with the loss of the endocrine, immunometabolic, and vascular competence of the adipose compartment, which is transformed from an adaptive energy depot into an active source of cardiorenometabolic dysfunction [31,32].

In particular, when visceral and ectopic adipose tissue exceed their capacity for adaptive expansion, a condition of adiposopathy develops, characterized by adipocyte hypertrophy, tissue hypoxia, macrophage infiltration, adipokine dysregulation, increased lipolysis, and greater release of free fatty acids into the portal and systemic circulation [33,34,35]. These processes promote hepatic and peripheral insulin resistance, sustain chronic low-grade inflammation, and contribute to the abnormalities in glucose and lipid metabolism that characterize the early stages of CKM [28,36,37,38]. In parallel, the reduction in adipokines with protective functions, such as adiponectin, and the increase in proinflammatory and profibrotic mediators promote endothelial dysfunction, oxidative stress, and vascular and myocardial remodeling, confirming that dysfunctional adipose tissue acts as an endocrinologically and immunologically active organ rather than as a mere compartment for energy storage [31,32,39,40].

Within the CKM continuum, these alterations do not remain confined to the metabolic domain alone. Dysfunctional adiposity promotes not only arterial hypertension, glucose dysmetabolism, and metabolic dysfunction-associated steatotic liver disease, but also glomerular hyperfiltration, early vascular damage, and progressive cardiorenometabolic vulnerability, even in the initial absence of overt diabetes, chronic nephropathy, or clinically evident heart disease [13,41]. In this context, activation of the renin–angiotensin–aldosterone system and the sympathetic nervous system, together with lipotoxicity and persistent systemic inflammation, contributes to integrating hemodynamic, metabolic, and fibrotic alterations that predispose to multiorgan damage. It follows that stage 1 of the CKM framework should not be interpreted as a merely preclinical phase or one quantitatively defined by body weight, but rather as a condition that is already biologically relevant, in which the functional imbalance of adipose tissue initiates the progressive transition from metabolic risk to renal and cardiovascular involvement [13,16,29].

Against this background, the limitation of body mass index as the sole indicator of risk becomes particularly evident, as it does not adequately distinguish fat distribution, lipid ectopia, or the biological quality of adipose tissue [34,42]. Within CKM pathophysiology, the value of dysfunctional adiposity lies precisely in making visible a biologically active condition capable of linking, through endocrine, inflammatory, hemodynamic, and metabolic mechanisms, the early alterations of the cardiometabolic profile with the subsequent development of renal damage, cardiovascular remodeling, and disease progression [43,44].

### 4.2. MASLD as the Hepatic Component of the CKM Continuum

Metabolic dysfunction-associated steatotic liver disease (MASLD) should not be considered an accessory hepatic comorbidity, but rather a pathophysiological component of the cardiovascular–kidney–metabolic continuum [13,29].

The terminological shift from non-alcoholic fatty liver disease to MASLD has made the metabolic basis of hepatic steatosis more explicit, repositioning liver disease within a systemic framework shared with dysfunctional adiposity, type 2 diabetes mellitus, chronic kidney disease, and cardiovascular disease [12,13,29,45,46].

From this perspective, MASLD belongs to the CKM continuum not only because of its frequent clinical co-occurrence, but also because it represents one of the main biological nodes through which dysmetabolism translates into multiorgan damage [12,13,29,45,46].

At the pathophysiological level, MASLD represents the interface between excess lipid flux to the liver, insulin resistance, lipotoxicity, oxidative stress, and the intrahepatic inflammatory response. Hepatic triglyceride accumulation is not a biologically neutral phenomenon, but is associated with mitochondrial dysfunction, altered fatty acid metabolism, increased gluconeogenesis, and worsening hepatic and peripheral insulin resistance. This gives rise to a self-amplifying circuit in which the steatotic liver is not merely a target organ of metabolic dysfunction, but also an effector organ capable of contributing to the propagation of systemic damage [29,47,48].

This active role is realized through continuous cross-talk with adipose tissue, endothelium, kidney, and myocardium. MASLD is closely intertwined with dysfunctional adiposity and chronic low-grade inflammation, contributing to alterations in lipid and lipoprotein metabolism, sustaining systemic lipotoxicity, and modulating, through hepatic endocrine and paracrine mediators, processes relevant to cardiorenal homeostasis. In this context, the liver participates in the systemic vulnerability of the CKM continuum not as an accessory element, but as a biological node that amplifies metabolic, inflammatory, and vascular signals already active in the early stages of disease [29,35,47,48].

From a clinical-pathophysiological perspective, the relevance of MASLD lies in the fact that its presence is associated with a more unfavorable cardiorenal profile, including a higher prevalence of subclinical atherosclerosis, alterations in cardiac function, increased risk of heart failure, albuminuria, reduced estimated glomerular filtration rate, and a greater likelihood of progression to chronic kidney disease [48,49,50].

These associations do not justify a rigidly causal interpretation in every individual patient, but they are consistent with the hypothesis that the hepatic component contributes to progression of the CKM continuum through shared mechanisms of insulin resistance, systemic inflammation, oxidative stress, and endothelial dysfunction [48,49,50].

Within this spectrum, metabolic dysfunction-associated steatohepatitis (MASH) may be cautiously considered the inflammatory and more progressive phenotype of MASLD, indicative of greater disease activity, without requiring an autonomous hepatological discussion in the present context. For these reasons, within the CKM framework, MASLD should be interpreted as the hepatic component of the continuum and as an expression of a shared inter-organ pathophysiology, rather than as a simple epiphenomenon of metabolic syndrome [51,52,53,54,55].

### 4.3. The Kidney as a Sentinel Organ and Amplifier

Within the CKM continuum, the kidney occupies a central position because it intercepts cardiometabolic vulnerability at an early stage and, in more advanced phases, actively contributes to the progression of multiorgan damage [13,18].

Rather than being merely a target organ, it is configured as a pathophysiological node in which dysfunctional adiposity, arterial hypertension, insulin resistance, hyperglycemia, endothelial dysfunction, and systemic hemodynamic alterations converge. In this early phase, glomerular hyperfiltration and increased intraglomerular pressure represent apparently compensatory adaptations, but their persistence promotes damage to the filtration barrier, endothelial stress, and progressive glomerular vulnerability [56,57].

In this context, albuminuria and estimated glomerular filtration rate (eGFR) acquire a significance that goes beyond the nephrological definition of chronic kidney disease alone [58].

Albuminuria represents one of the earliest signals of renal involvement in the CKM continuum and reflects, at the same time, a broader systemic endothelial dysfunction; for this reason, it is consistently associated with increased cardiovascular risk, heart failure, progression of chronic kidney disease, and all-cause mortality [15,58,59,60]. Complementarily, reduced eGFR not only documents loss of renal function, but also identifies a phenotype with high cardiorenal vulnerability. It is precisely the structural inclusion of albuminuria and eGFR that constitutes one of the elements making the CKM framework prognostically more informative than traditional metabolic syndrome, because it allows early recognition of organ damage that is often already clinically relevant before the onset of overt cardiovascular disease [6,19].

Once renal dysfunction becomes established, the kidney ceases to be merely an early sentinel and becomes an amplifier of the cardiorenometabolic trajectory. Reduced renal functional reserve promotes sodium and water retention, congestion, activation of the renin–angiotensin–aldosterone system, and sympathetic overactivation, thereby fueling a self-reinforcing circuit of vasoconstriction, increased afterload, inflammation, fibrosis, and myocardial and vascular remodeling [11,61,62]. Additional alterations in systemic biology are also associated with this process, including anemia, disorders of mineral metabolism, and metabolic acidosis, all of which contribute to worsening arterial stiffness, myocardial vulnerability, and progression of organ damage [63]. The kidney therefore participates in the CKM continuum not only as a site of damage, but also as an organ that translates and amplifies hemodynamic, neurohormonal, inflammatory, and metabolic signals originating from other districts.

**Figure 4:**
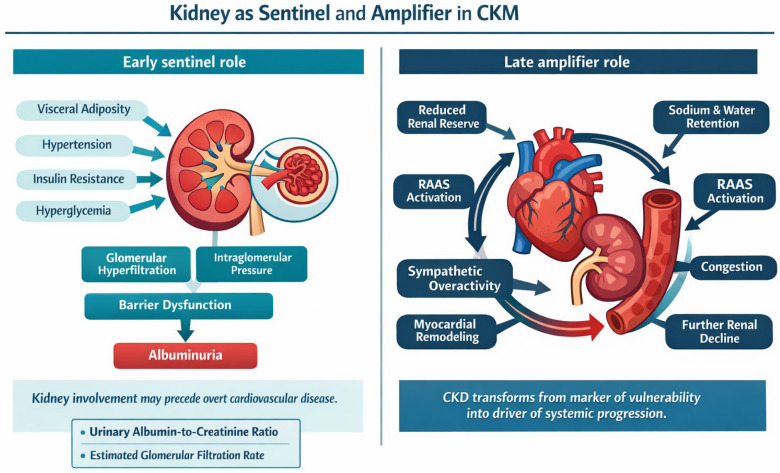
Kidney as sentinel and amplifier in cardiovascular–kidney–metabolic (CKM) syndrome. The figure highlights the kidney as an early marker of CKM injury and, in later stages, as a driver of cardio-renal amplification through progressive hemodynamic and neurohormonal dysfunction.

Figure 4 therefore summarizes the dual role of the kidney in CKM, showing how it initially acts as an early sentinel of organ vulnerability through hyperfiltration and albuminuria and, subsequently, as an amplifier of systemic cardiorenal progression through neurohormonal activation, congestion, and further functional decline.

This interpretation is particularly relevant because it clarifies how renal involvement may precede clinically manifest heart disease and, subsequently, substantially influence the patient’s overall prognostic profile [15,56]. On the one hand, alterations such as glomerular hyperfiltration and albuminuria often anticipate more advanced cardiovascular phenotypes; on the other hand, once chronic kidney disease has become established, cardiovascular risk increases markedly and progressively, delineating a phenotype of high biological and clinical complexity in which renal impairment does not represent an epiphenomenon, but rather a major determinant of multiorgan progression [60,64].

Within the CKM model, the kidney should therefore be regarded both as an early sentinel of systemic vulnerability and as an amplifier of cardiorenometabolic progression, with a pathophysiological and prognostic value that fully justifies the central role of eGFR and albuminuria in integrated risk stratification [13,18,62].

### 4.4. Cardiovascular Manifestations of the CKM Continuum

Within the CKM continuum, the cardiovascular domain does not represent an isolated terminal outcome of metabolic risk, but rather a structural component of the disease trajectory, developing progressively through the interaction among dysfunctional adiposity, insulin resistance, chronic low-grade inflammation, endothelial dysfunction, neurohormonal activation, and renal damage [13,65]. Cardiovascular manifestations are therefore not limited to atherosclerotic disease alone, but encompass a broader spectrum of vascular and cardiac abnormalities, both subclinical and clinically manifest, reflecting the multiorgan nature of the CKM framework [6,42,66,67].

In the preclinical phases of the continuum, cardiovascular involvement may be expressed through endothelial dysfunction, arterial stiffness, left ventricular remodeling, abnormalities of diastolic function, microvascular disease, and other forms of subclinical cardiovascular damage, which often precede major events while anticipating their risk profile [8,13].

In this sense, the subclinical phase does not constitute an intermediate zone devoid of clinical significance, but rather a stage of progressive organ translation of cardiorenometabolic vulnerability, in which pressure overload, lipotoxicity, systemic inflammation, and hemodynamic alterations converge [8,13,67,68].

The value of these manifestations lies precisely in the fact that they document an already established progression of the continuum, making the transition from risk factors to organ damage and overt cardiovascular disease more readily discernible [6,18].

Alongside the atherosclerotic component, driven by atherogenic dyslipidemia, insulin resistance, glucotoxicity, vascular inflammation, and thrombo-inflammatory activation, the CKM continuum also includes forms of cardiac remodeling not attributable exclusively to coronary ischemia [42,66,69].

The myocardium of the patient with CKM is simultaneously exposed to pressure and volume overload, alterations in energy metabolism, ectopic fat accumulation, inflammation, and microvascular dysfunction, in a context that is further aggravated, in more advanced stages, by renal impairment and congestion [68,70,71]. These processes promote ventricular hypertrophy, increased wall stiffness, altered ventriculo-arterial coupling, and reduced functional reserve, thereby creating a particularly favorable substrate for the development of heart failure, especially in phenotypes with preserved ejection fraction [68,72,73]. From this perspective, obesity-associated heart failure with preserved ejection fraction (HFpEF) represents one of the most paradigmatic expressions of the cardiorenometabolic continuum, because it makes clinically evident the convergence among dysfunctional adiposity, systemic inflammation, microvascular damage, cardiac remodeling, and cardiorenal interdependence [73,74].

Even when atherosclerotic disease is not the predominant mechanism, cardiovascular involvement remains deeply rooted in the same integrated pathophysiology that underlies metabolic and renal progression. For this reason, within the CKM framework, cardiovascular manifestations should be interpreted not only as final complications, but as progressive and mutually reinforcing expressions of a multiorgan vulnerability, in which subclinical damage and heart failure carry prognostic relevance no less important than that of overt atherosclerotic disease [6,8,18].

The main pathophysiological nodes and their inter-organ effects across the CKM continuum are summarized in Table 3.

## 5. Social Determinants of Health and the Life-Course Perspective

Within the CKM continuum, the social determinants of health are not merely contextual variables, but upstream factors that contribute to the distribution of risk across all stages of the disease [75,76].

Conditions such as socioeconomic disadvantage, food insecurity, reduced access to care, job insecurity, and housing instability may promote early entry into the initial stages of the continuum by fostering dysfunctional adiposity, dysmetabolism, arterial hypertension, and chronic kidney disease at moderate or high risk.

From this perspective, CKM makes clinically visible the way in which social inequalities translate into a concrete acceleration of cardiorenometabolic vulnerability [75,76,77].

This relevance is not merely descriptive. In population analyses, adverse social determinants are associated with a higher prevalence of advanced CKM stages, with unfavorable gradients becoming more evident when multiple conditions of disadvantage coexist, such as low income, unemployment, food insecurity, and reduced access to healthcare services. The result is a progressive widening of the risk base, which over time feeds the pool of individuals destined to progress toward subclinical cardiovascular disease, high-risk equivalents, or clinically manifest disease [21,77,78].

The life-course perspective helps interpret this process as an accumulation of vulnerability rather than as the simple late appearance of comorbidities. Indeed, CKM risk does not depend exclusively on the biological burden present at the time of assessment, but also on the duration and repetition over time of adverse exposures that interact with metabolic predisposition, environment, health behaviors, and opportunities for prevention [79,80].

In this way, social determinants influence not only the early onset of disease, but also the speed of progression and the point at which the continuum is actually intercepted by the healthcare system. Integrated pathophysiology explains why the heart, kidney, liver, and adipose tissue are damaged in a coordinated manner; the life-course perspective explains why this process begins earlier in some individuals, progresses more rapidly, and is recognized later [16,76,81,82,83].

From a clinical and organizational standpoint, the relevance of social determinants emerges at least at three critical points along the care pathway: early disease onset, progression due to suboptimal control of risk factors, and prognosis, through diagnostic delays, reduced access to innovative treatments, lower continuity of care, and poorer therapeutic adherence. For this reason, risk stratification that is truly consistent with the CKM model cannot be limited to biological markers and organ damage, but must also include the factors that modulate the actual possibility of prevention, early diagnosis, and effective treatment. This does not mean shifting the focus from clinical medicine to social medicine, but rather recognizing that, in CKM, these two dimensions are closely intertwined and jointly shape outcomes. In this sense, the social determinants of health represent a necessary element for understanding the real usefulness of the CKM framework in contemporary clinical practice and naturally set the stage for its implementation-related implications [13,84,85].

## 6. Clinical Implications of the CKM Model

The main clinical utility of the cardiovascular–kidney–metabolic framework does not lie in the introduction of a new nosological label, but in its ability to provide more precise risk stratification than models based exclusively on traditional metabolic risk factors [6]. In particular, CKM makes it possible to integrate into a single prognostic framework dysfunctional adiposity, diabetes or prediabetes, chronic kidney disease, subclinical cardiovascular disease, and the clinical-social context, thereby overcoming the limitations of metabolic syndrome as a predominantly descriptive construct [2,8]. Proper stratification first requires the definition of the CKM stage, but staging should not be interpreted as a self-sufficient classification [13]. In clinical practice, it acquires value above all when integrated with a more refined phenotyping approach, based on the patient’s position along the disease continuum, the predominant axis of organ damage, and the intensity of intervention required at that stage [16,18].

**Figure 5:**
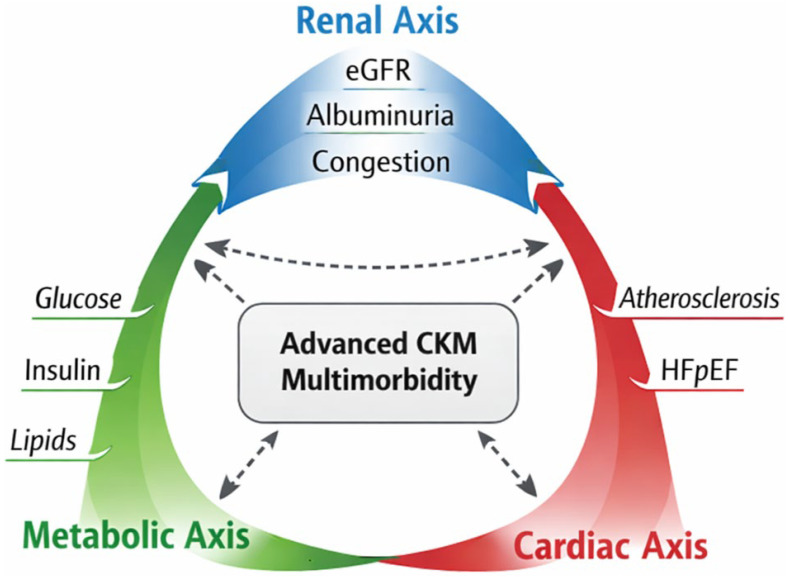
Tri-axial representation of advanced cardiovascular–kidney–metabolic (CKM) multimorbidity. The figure emphasizes how advanced CKM arises from reciprocal dysfunction across the metabolic, renal, and cardiac axes rather than from isolated organ-specific disease.

As shown in Figure 5, advanced CKM may be interpreted as a condition of integrated multimorbidity, sustained by the interaction among the metabolic, renal, and cardiac axes. This representation underscores that risk does not depend on isolated individual domains, but on their reciprocal clinical and pathophysiological interdependence.

From this perspective, one of the main advantages of the CKM model is the systematic inclusion of the renal axis in prognostic assessment [6,19]. Indeed, the combination of estimated glomerular filtration rate (eGFR) and the urinary albumin-to-creatinine ratio makes it possible to detect early renal damage, substantially refine cardiovascular risk, and identify highly vulnerable phenotypes that metabolic syndrome alone tends to underestimate [9,15,86,87,88]. Similarly, the presence of subclinical cardiovascular disease or high-risk equivalents allows recognition of an intermediate phase of major clinical relevance, in which intensive prevention may still modify the disease trajectory [8,89,90,91]. In the European context, this stratification logic must also be aligned with locally validated predictive tools [92,93]. The Predicting Risk of Cardiovascular Disease Events (PREVENT) equations represent a conceptual advance because they broaden cardiovascular risk assessment beyond atherosclerotic events alone, also including heart failure [8,25]. However, because they were derived from US cohorts, their application to European and Italian populations requires interpretive caution; the value of the CKM framework therefore lies primarily in enabling the integration of such tools with indicators of organ damage and with multidimensional clinical assessment, rather than in proposing a single universal algorithm [13].

From a practical standpoint, the CKM model promotes a less fragmented approach to the care of patients with cardiorenometabolic multimorbidity, encouraging an integrated interpretation of the metabolic, renal, and cardiovascular domains and making recourse to multidisciplinary co-management more coherent in the most complex phenotypes [6,8]. In pragmatic terms, a minimum clinical dataset, rationally constructed for the assessment of cardiovascular–kidney–metabolic syndrome, should include weight history and adiposity distribution, blood pressure and hemodynamic profile, glycometabolic and lipid profile, renal function with estimation of glomerular filtration rate and albuminuria, clinical or instrumental evidence of subclinical or overt cardiovascular damage, the presence and severity of metabolic dysfunction-associated steatotic liver disease (MASLD), associated comorbidities, ongoing therapies and their tolerability, lifestyle habits, and the main social determinants of health, including access to care, therapeutic adherence, and psychosocial context [6,45]. This proposal should be understood as an operational synthesis of the main clinical domains useful for applying the CKM model in real-world practice, and not as an official dataset or one formally standardized by the American Heart Association.

Within this framework, the potential to slow progression across the CKM continuum depends less on isolated disease labels than on timely, stage-based intervention. In stages 1–2, the greatest opportunity lies in early recognition of dysfunctional adiposity, metabolic abnormalities, and incipient renal involvement, coupled with lifestyle optimization and rigorous control of modifiable risk factors before fixed multiorgan damage becomes established [6,8,18]. In subsequent stages, clinical evaluation should become progressively more refined through systematic assessment of albuminuria, estimated glomerular filtration rate, and subclinical cardiovascular involvement, so that risk is not only classified but more accurately redefined according to evolving organ vulnerability [8,9,15,18]. In more complex phenotypes, the practical value of the CKM model lies in supporting cross-domain strategies that integrate metabolic, renal, and cardiovascular priorities rather than addressing them sequentially or in parallel silos [6,13,16]. From this perspective, multidisciplinary care is relevant not because CKM requires a new therapeutic doctrine, but because coordinated use of established organ-protective approaches may be most effective when multimorbidity has already become clinically interconnected.

However, it remains necessary to maintain a cautious formulation regarding the possible operational implications of the framework. CKM may offer a clinically useful framework for guiding the intensity of assessment, promoting early recognition of organ damage, and supporting stage-based management that is more consistent with real-world multimorbidity; nonetheless, it does not in itself coincide with an exhaustive guideline, nor does it automatically define universal monitoring protocols or rigidly standardized care pathways [6,13]. Its use therefore appears particularly valuable as an interpretive and organizational structure capable of bringing cardiology, nephrology, diabetology, metabolic hepatology, and internal medicine closer together, but its implementation must remain contextualized to different clinical settings, available resources, and predictive tools validated within individual healthcare systems [8,92,93].

The proposed minimum clinical dataset and its practical domains of application are detailed in Table 4.

## 7. Limitations of the CKM Model

The cardiovascular–kidney–metabolic syndrome framework represents a relevant conceptual advance over traditional metabolic syndrome, but its value can be appropriately interpreted only when placed within an explicit critical framework [6]. In particular, its usefulness does not justify an overly assertive interpretation of CKM staging, especially when it is used as the basis for epidemiological comparisons or for generalized inferences across different populations [17,18]. CKM, in fact, organizes already known and closely interconnected conditions along a continuum, offering a more coherent framework for interpreting cardiorenometabolic multimorbidity; however, like any model of synthesis, it simplifies a biological and clinical reality that remains heterogeneous, dynamic, and often non-linear [13,94].

A first limitation is epidemiological in nature. CKM does not identify a single disease with autonomous diagnostic criteria, but rather reclassifies pathological domains whose prevalence depends on the operational definitions adopted, the characteristics of the cohorts, and the intensity of screening for the different components of the continuum [6,21]. It follows that the distribution of stages observed in US cohorts cannot be automatically transferred to other geographical, healthcare, or socioeconomic settings [18,21]. Differences in the prevalence of obesity, diabetes, chronic kidney disease, MASLD, and access to care may substantially modify both the composition of the stages and their prognostic significance [22,23,24]. For this reason, the framework still requires more robust external validation in European and non-North American populations, as well as local calibration of the predictive tools associated with it [8].

A second limitation concerns the relationship between classificatory simplicity and the complexity of the real-world patient. CKM staging improves the readability of progression from metabolic risk to overt cardiorenal damage, but it does not exhaust the clinical variability that characterizes individual phenotypes [13,94]. Patients classified within the same stage may differ substantially in biological age, disease duration, distribution and quality of adiposity, degree of inflammation, severity of MASLD, frailty, polypharmacy, and social context [27,29]. Consequently, staging cannot be regarded as a substitute for individual clinical judgment, nor as a system already universally validated for all diagnostic, prognostic, or therapeutic decisions [19]. The strength of the model therefore does not lie in entirely replacing previous categories, but in providing a shared language better suited to integrating metabolic factors, renal damage, cardiovascular risk, and social determinants into a unified reading of the patient [6]. In this sense, CKM should be interpreted as a guiding structure rather than as a definitive taxonomy [17].

A third limitation is implementational and organizational in nature. Consistent application of the CKM framework presupposes an integrated model of care capable of linking cardiology, nephrology, diabetology, hepatology, internal medicine, and the social determinants of health within shared pathways [6]. In practice, however, many healthcare systems remain organized in specialist silos, with fragmented access, data that are not always interoperable, variable continuity of care, and incomplete standardization of certain practical aspects. In this context, the risk is that CKM may be recognized as a theoretically convincing paradigm but applied heterogeneously across centers, with a consequent reduction in reproducibility and clinical impact. Its usefulness will therefore also depend on the ability to translate the classification into concrete, sustainable, and measurable models of care [95]. Overall, these limitations do not diminish the value of the CKM framework, but rather define its proper interpretive boundaries: a useful and evolving clinical-prognostic framework whose application requires methodological caution, adaptation to context, and further operational consolidation.

## 8. Future Perspectives

The future prospects of the CKM framework will depend first and foremost on its ability to consolidate itself as a more inclusive risk stratification tool without losing methodological rigor. From this perspective, the evolution of predictive equations represents an important step forward: tools such as the PREVENT equations may contribute to an interpretation more consistent with the cardiorenometabolic continuum, as they broaden risk assessment beyond atherosclerotic events alone. However, their application outside US cohorts still requires external validation and local calibration; in particular, in the European context, such models will need to be integrated with already established risk scores rather than automatically replacing them [8,25,96,97,98].

A second area of development concerns the progressive convergence between the CKM paradigm and clinical approaches capable of acting simultaneously on multiple risk domains. In this sense, the growing availability of interventions with cross-domain effects on cardiovascular risk, progression of chronic kidney disease, dysfunctional adiposity, and metabolic control makes the framework particularly relevant, not so much as an exhaustive therapeutic container, but rather as a useful framework for guiding earlier and less fragmented decisions along the disease trajectory [99,100,101]. In parallel, the consolidation of MASLD as an operational component of the continuum may contribute to a more complete phenotyping of systemic vulnerability, especially in patients in whom the steatotic liver signals a biologically active phase of metabolic dysfunction and cardiorenal risk [12,45,102,103].

More generally, the future of the model will depend on its ability to remain essential, standardizable, and translatable across different care settings. Because CKM spans multiple specialist domains, its implementation will require models of care capable of synthesis, longitudinality, and prioritization, as well as contextual validation to clarify its transferability and practical usefulness in different populations. From this perspective, the most promising trajectory is not that of indefinitely expanding the paradigm, but rather of making it more precise, better validated, and more useful for the prevention and real-world management of cardiorenometabolic multimorbidity [6,25,96,97,98].

Ultimately, the ability of the CKM framework to modify disease trajectory and prognosis will depend on how effectively risk stratification is coupled with early, integrated intervention across the continuum.

## Figures and Tables

**Table 1:** From Metabolic Syndrome to CKM: conceptual differences and added clinical value.

Dimension	Metabolic Syndrome	CKM Framework	Added Clinical Value	References
Conceptual focus	Cluster of cardiometabolic risk factors used pragmatically for risk communication	Unified clinical-prognostic framework linking adiposity, metabolic dysfunction, kidney disease, and cardiovascular disease	Shifts the focus from factor clustering to integrated multiorgan risk interpretation	[1,2,6,13]
Disease model	Predominantly descriptive construct	Dynamic continuum from early dysmetabolism to overt renal and cardiovascular organ damage	Better reflects progression rather than a static coexistence of abnormalities	[6,13]
Organ involvement	Mainly centered on visceral adiposity, blood pressure, lipids, and glucose metabolism	Explicitly incorporates kidney involvement, subclinical and clinical cardiovascular disease, and MASLD within the same continuum	Broadens clinical reading of cardiorenometabolic multimorbidity	[1,2,6,8,9,11,12]
Risk interpretation	Identifies subjects at increased risk of diabetes and cardiovascular disease	Reinterprets risk as a shared prognostic trajectory shaped by interdependent metabolic, renal, and cardiovascular domains	Provides a more biologically coherent and clinically realistic reading of vulnerability	[1,2,6,8,13]
Renal dimension	Not structurally centered on renal markers	Integrates eGFR and albuminuria into risk definition and stratification	Enables earlier recognition of clinically relevant organ damage and refines prognostic assessment	[8,9,13]
Cardiovascular dimension	Mainly oriented toward future cardiovascular risk in broad terms	Includes subclinical cardiovascular disease and heart failure, not only atherosclerotic disease	Improves recognition of intermediate and advanced stages of disease progression	[6,8,9]
Longitudinal view	Does not frame patients within an explicit stage-based continuum	Places the patient along a potentially modifiable stage-based trajectory over time	Supports a more progressive and longitudinal interpretation of disease evolution	[8,9,13]
Contextual and life-course perspective	Largely absent from the traditional construct	Explicitly acknowledges cumulative exposures, life transitions, and social determinants of health	Makes risk assessment more consistent with real-world heterogeneity and progression	[6,8,10,13]
Practical clinical usefulness	Useful for identifying clustered metabolic risk	Useful for reorganizing multiorgan risk without claiming to be a new autonomous disease or a self-sufficient algorithm	Offers a more integrated framework for risk stratification while remaining compatible with clinical judgment and validated tools	[6,8,17,19,20]

CKM: Cardiovascular–kidney–metabolic; MASLD: Metabolic dysfunction-associated steatotic liver disease; eGFR: estimated glomerular filtration rate.

**Table 2:** Epidemiological and prognostic reading of the CKM continuum.

Epidemiological/Prognostic Domain	Key Message from the Review	Clinical/Public Health Implication	Interpretive Caution Or Limitation	References
Nature of CKM epidemiology	CKM should be read as an integrated framework that reorganizes highly prevalent and overlapping metabolic, renal, and cardiovascular conditions, rather than as a new autonomous disease entity.	Supports a multiorgan reading of multimorbidity and helps interpret burden as a continuum rather than as isolated disorders.	Epidemiological estimates reflect the way existing conditions are clustered and staged, not the incidence of a distinct nosological entity.	[6,19,20]
Main source of current staging data	The most direct estimates of CKM burden come from application of CKM staging to US adults in NHANES 2011–2020.	Provides an initial population-level picture of how the continuum is distributed across stages.	Current staging estimates are derived mainly from US cohorts and should not be generalized without external validation.	[10,20]
Stage distribution and burden concentration	Only a minority of adults are classified as stage 0, whereas most burden is concentrated in stages 1–2; a non-negligible proportion is already in stages 3–4.	Indicates that much of the burden lies in early or intermediate phases, before overt cardiovascular disease, with relevant implications for prevention and reclassification.	Stage distribution depends on how stages are operationalized and on availability of renal and subclinical cardiovascular assessments.	[9,21]
Public health meaning of stages 1–2	Early and intermediate stages already include excess or dysfunctional adiposity, metabolic risk factors, and in stage 2 also CKD at moderate-to-high risk.	These stages are highly relevant because they represent a large pool of individuals in whom progression may still be modified through earlier recognition and prevention.	Their apparent prevalence may vary according to screening intensity and cohort characteristics.	[8,9,10,21]
Prognostic meaning of CKD, eGFR, and albuminuria	CKD, reduced eGFR, and albuminuria identify phenotypes at higher risk than suggested by a purely factor-centered interpretation.	Renal markers add prognostic depth and help identify clinically relevant vulnerability before overt cardiovascular events.	Their contribution to stage assignment and risk interpretation depends on consistent measurement and on the definitions adopted.	[8,10,19,21,25]
Relevance of subclinical cardiovascular disease	Subclinical cardiovascular disease contributes substantially to CKM burden by identifying a transition from risk-factor clustering to organ damage.	Improves prognostic stratification and highlights patients already on a more advanced trajectory despite absence of overt clinical events.	Detection is influenced by the availability and intensity of instrumental screening across settings.	[8,10,19,21,25]
Global burden convergence of obesity, diabetes, and CKD	Global trends show rising burden of overweight/obesity, diabetes, and CKD, reinforcing the epidemiological relevance of CKM as a convergent multiorgan continuum.	CKM is useful because it frames these common conditions as interacting drivers of shared long-term burden, not as separate epidemics only.	Global component trends do not automatically define uniform CKM-stage distributions across regions or systems.	[22,23,24]
Dependence on operational definitions and screening intensity	The prevalence of CKM stages depends on operational criteria, cohort composition, and the availability of measures such as eGFR, albuminuria, and markers of subclinical cardiovascular damage.	Reminds clinicians and researchers that staging-based burden estimates are method-dependent.	Comparisons across studies or settings may be distorted if ascertainment strategies differ.	[8,21]
Transferability across settings	US-derived estimates cannot be automatically transferred to other geographical or healthcare contexts.	Encourages context-aware interpretation of stage prevalence and prognostic meaning.	Differences in adiposity, diabetes, CKD, MASLD, access to care, and healthcare structure may alter both distribution and meaning of stages.	[8,10,19,21,25]
Need for external validation and local calibration	Broader validation in non-North American populations and local calibration of predictive tools are needed to preserve the robustness of the model.	Necessary before CKM-based epidemiological or prognostic tools are applied broadly across different populations.	Without validation and calibration, inappropriate generalization may weaken interpretive reliability.	[8,25,26]

CKD: chronic kidney disease; CKM: Cardiovascular–kidney–metabolic; eGFR: estimated glomerular filtration rate; MASLD: Metabolic dysfunction-associated steatotic liver disease; NHANES: National Health and Nutrition Examination Survey; US: United States.

**Table 3:** Integrated pathophysiology of CKM across adipose tissue, liver, kidney and cardiovascular system.

Biological Node/Organ Domain	Main Pathophysiological Mechanisms	Systemic/Inter-Organ Effects	Role in CKM Progression	References
Dysfunctional adiposity	Loss of endocrine, immunometabolic, and vascular competence of adipose tissue; adipocyte hypertrophy, tissue hypoxia, macrophage infiltration, adipokine dysregulation, increased lipolysis, increased free fatty acid flux	Promotes hepatic and peripheral insulin resistance, chronic low-grade inflammation, endothelial dysfunction, oxidative stress, vascular and myocardial remodeling; contributes to hypertension, MASLD, glomerular hyperfiltration, and early vascular damage	Biological trigger of the continuum; transforms early adiposity-related risk into multiorgan vulnerability even before overt diabetes, CKD, or clinical heart disease	[13,28,31,32,33,34,35,36,37,38,39,40,41,43,44]
Insulin resistance/metabolic-inflammatory hub	Shared disturbance linking adiposity, altered glucose and lipid metabolism, lipotoxicity, oxidative stress, neurohormonal activation, and fibroinflammatory remodeling	Connects adipose tissue, liver, kidney, vasculature, and myocardium through self-amplifying metabolic-inflammatory circuits rather than isolated lesions	Central integrative hub that sustains transition from metabolic-inflammatory vulnerability to subclinical organ damage across CKM domains	[13,17,27,28,29,36,37,38,47,48]
MASLD/hepatic component	Excess lipid flux to the liver, hepatic triglyceride accumulation, insulin resistance, lipotoxicity, oxidative stress, mitochondrial dysfunction, altered fatty acid metabolism, increased gluconeogenesis, intrahepatic inflammation	Steatotic liver acts not only as target but also as effector organ; amplifies systemic dysmetabolism, lipotoxicity, inflammatory and vascular signaling through cross-talk with adipose tissue, endothelium, kidney, and myocardium	Hepatic biological node of CKM, contributing to propagation of systemic damage and to a less favorable cardiorenal profile	[12,13,29,45,46,47,48,49,50,51,52,53,54,55]
Kidney as early sentinel	Convergence of dysfunctional adiposity, hypertension, insulin resistance, hyperglycemia, endothelial dysfunction, and hemodynamic stress; glomerular hyperfiltration, increased intraglomerular pressure, barrier dysfunction, albuminuria	Early renal abnormalities signal broader endothelial and microvascular dysfunction; eGFR reduction and albuminuria refine cardiovascular and renal risk before overt CVD	Sentinel organ that detects early organ vulnerability and makes CKM risk clinically visible at a pre-overt stage	[6,13,15,18,19,56,57,58,59,60]
Kidney as late amplifier	Reduced renal reserve, sodium and water retention, congestion, RAAS activation, sympathetic overactivity; associated anemia, mineral metabolism disorders, and metabolic acidosis	Amplifies vasoconstriction, afterload, inflammation, fibrosis, myocardial and vascular remodeling, and further renal decline	Converts renal involvement from early marker into active driver of systemic cardiorenal progression	[11,13,18,61,62,63,64]
Vascular dysfunction and subclinical cardiovascular damage	Endothelial dysfunction, arterial stiffness, microvascular disease, pressure overload, lipotoxicity, systemic inflammation, hemodynamic alterations	Represents translation of metabolic and renal vulnerability into progressive organ damage; anticipates major cardiovascular events and documents established continuum progression	Intermediate but clinically relevant phase linking risk factors to overt cardiovascular disease	[6,8,13,18,65,66,67,68]
Myocardial remodeling/HF-prone phenotype	Combined pressure and volume overload, altered energy metabolism, ectopic fat accumulation, inflammation, microvascular dysfunction, renal impairment, congestion	Promotes ventricular hypertrophy, increased wall stiffness, altered ventriculo-arterial coupling, reduced functional reserve, and a substrate favorable to heart failure, especially HFpEF-related phenotypes	Cardiac expression of integrated CKM biology, not limited to ischemic disease; highlights heart failure as a central manifestation of the continuum	[42,66,68,70,71,72,73,74]
Self-reinforcing multiorgan feedback circuits	Shared dynamic circuits of inflammation, oxidative stress, endothelial dysfunction, neurohormonal activation, lipotoxicity, and fibroinflammatory remodeling operating across adipose tissue, liver, kidney, vasculature, and myocardium	Bidirectional cross-talk among organs perpetuates dysmetabolism, early renal damage, vascular injury, and cardiac remodeling	Explains why CKM is an integrated biological condition rather than the sum of separate comorbidities, and why progression is network-based rather than rigidly linear	[13,16,17,19,27,28,29,65]

CKD: chronic kidney disease; CKM: Cardiovascular–kidney–metabolic; CVD: cardiovascular disease; eGFR: estimated glomerular filtration rate; HFpEF: heart failure with preserved ejection fraction; MASLD: Metabolic dysfunction-associated steatotic liver disease; RAAS: renin–angiotensin–aldosterone system.

**Table 4:** Minimum clinical dataset and practical domains for CKM assessment.

Clinical Domain	Minimum Elements to Assess	Why it Matters in CKM	Practical Contribution to Integrated Evaluation	References
Anthropometry and adiposity profile	Weight history; adiposity distribution	Dysfunctional adiposity is an early driver of the continuum and may signal biologically relevant risk beyond a simple descriptive metabolic cluster	Helps position the patient along the continuum from early vulnerability to more advanced multiorgan involvement; supports phenotyping beyond isolated diagnoses	[6,13,16,18]
Blood pressure and hemodynamic profile	Blood pressure; overall hemodynamic profile	Hemodynamic load interacts with metabolic dysfunction and renal vulnerability, contributing to progression across CKM domains	Refines interpretation of vascular and cardiorenal burden within a multidimensional assessment	[6,8,13]
Glycemic and lipid-metabolic domain	Glycometabolic profile; lipid profile; presence of diabetes or prediabetes	Dysmetabolism remains a core axis of CKM and contributes to transition from metabolic risk to organ damage	Supports stage definition and integrated phenotyping of the metabolic component of multimorbidity	[2,6,8,13]
Renal assessment	Renal function with estimation of glomerular filtration rate; albuminuria	The renal axis is a distinctive prognostic component of CKM; early renal damage may identify vulnerable phenotypes underestimated by metabolic syndrome alone	Improves risk refinement, detects early organ involvement, and anchors multiorgan assessment to cardiorenal vulnerability	[6,9,15,19,86,87,88]
Cardiovascular assessment	Clinical or instrumental evidence of subclinical or overt cardiovascular damage	CKM explicitly includes subclinical cardiovascular disease and overt cardiovascular disease within the same continuum	Distinguishes intermediate versus advanced disease expression and improves staging-oriented interpretation of risk	[8,13,16,18,89,90,91]
Liver-related metabolic dysfunction	Presence and severity of MASLD	MASLD is integrated as a hepatic component of systemic metabolic dysfunction rather than an accessory comorbidity	Broadens CKM phenotyping and helps capture multiorgan burden more coherently	[45]
Comorbidities and multimorbidity profile	Associated comorbidities	CKM is clinically useful because it integrates coexisting metabolic, renal, cardiovascular, and related conditions within one framework	Prevents fragmented reading by single disease and supports recognition of the predominant axis of damage	[6,8,16,18]
Ongoing treatment and tolerability	Current therapies; tolerability	Clinical interpretation of CKM should be contextualized to the intensity of intervention required and to real-world patient complexity	Contributes to pragmatic phenotyping and to a realistic assessment of implementability in routine care	[13,16,18]
Lifestyle and behavioral domain	Lifestyle habits	CKM progression is shaped not only by biological damage but also by modifiable behavioral exposures	Adds practical context to risk interpretation and supports a less reductionist evaluation of disease trajectory	[6,8,13]
Social and contextual modifiers	Access to care; therapeutic adherence; psychosocial context; main social determinants of health	Social and contextual factors influence prevention, early diagnosis, and the actual possibility of effective care across the continuum	Completes multidimensional risk assessment by identifying factors that modulate staging expression, prognosis, and real-world vulnerability	[13,75,76,84,85]
Integrated risk interpretation	CKM stage integrated with patient position along the continuum, predominant axis of organ damage, and multidimensional clinical context	Staging alone is not self-sufficient; CKM has value when combined with refined phenotyping and validated tools interpreted within context	Synthesizes burden, organ involvement, and subclinical vulnerability into a clinically coherent cardiorenal-metabolic reading	[6,8,13,16,18,92,93]

CKM: Cardiovascular–kidney–metabolic; MASLD: Metabolic dysfunction-associated steatotic liver disease.

## Data Availability

No new data were created or analyzed in this study.
